# Disease in the Urinary Organs; Stone in the Kidney

**Published:** 1852-02

**Authors:** 


*Disease in the Urinary Organs; Stone in the Kidney.*—Mr. Coulson exhibited a specimen taken from a man, fifty-three years of age, who had suffered upwards of twenty years from stricture of the urethera, occasional abscess in the perinæum and retention of urine. On the 19th of September, he was admitted into St. Mary's Hospital; the urine passed, in drops, both through an aperture in the perinæum, and the urethra; it was alkaline, and contained a good deal of mucus and albumen; no instrument could be passed through the stricture, which was seated at the bulbous portion of the urethra. As the pain and difficulty of passing the urine were very great, it was thought that a free exit for the discharge of the water might lessen the patient's sufferings. On the 8th of October, Mr. Coulson divided the stricture through the perinæum, and easily introduced a catheter, which was retained in the bladder forty-eight hours. The first effect was relief to the pain, but on the next day it returned, and increased in severity until the 14th, when he died.

*Post-Mortem Examination.*—There was a wound an inch in length in the median line of the perinæum, dividing the bulbous portion of the urethra, which was considerably thickened. In the membranous part there were two openings, one leading to the abscess in the perinæum, and the other going towards the pelvis. The bladder was very much thickened, and its mucous membrane in several parts destroyed. On the posterior surface of the prostate there was an abscess about the size of a hen's egg, not communicating, however, either with the bladder or the rectum. The left kidney was enlarged and distended with pus, the whole of its proper structure being absorbed; in its lower part there was a large calculus, which occupied nearly the whole of the lower half of the kidney, projecting into the infundibulum and pelvis. The ureter was very much enlarged and thickened, the mucous membrane being rough and of a dark color about the centre; there was a contraction of the canal and hardness of its coats. The right kidney and other organs were healthy, with the exception of the liver, in which there was incipient fatty degeneration.—*Ibid.*

